# Motor, Postural, and Communicative Phenotype in SHQ1-Related Neurodevelopmental Disorder with Dystonia and Seizures: An Interdisciplinary Case Report and Hypothesis-Generating Physiotherapy Framework

**DOI:** 10.3390/jcm15197379

**Published:** 2026-09-23

**Authors:** Artur Polczyk, Natalia Banaszek-Hurła, Ewelina Wolańska, Joanna Wawrzyniak, Natalia Purpurowicz-Miękus, Anna Winczewska-Wiktor, Barbara Steinborn, Robert Śmigiel

**Affiliations:** 1 Medical Education and Simulation Laboratory, University Centre of Physiotherapy and Rehabilitation, Faculty of Physiotherapy, Wroclaw Medical University, 50-368 Wroclaw, Poland; e.wolanska@umw.edu.pl; 2 Department of Developmental Neurology, Poznan University of Medical Sciences, 60-355 Poznan, Poland; 3 Clinical Center of Gynecology, Obstetrics and Neonatology, 45-066 Opole, Poland; 4 Department of Pediatrics, Endocrinology, Diabetology and Metabolic Diseases, Wroclaw Medical University, 50-368 Wroclaw, Poland; robert.smigiel@umw.edu.pl

**Keywords:** SHQ1, NEDDS, developmental and epileptic encephalopathy, dystonia, spasticity, cerebellar atrophy, cannabidiol, augmentative and alternative communication, Bayley-4, Vojta-derived Locomotion Stages

## Abstract

**Background**: Biallelic variants in SHQ1 (SHQ1 homolog; H/ACA small nucleolar ribonucleoprotein assembly factor) cause neurodevelopmental disorder with dystonia and seizures (NEDDS), but descriptions of spontaneous motor organization, functional communication, and rehabilitation-relevant abilities remain limited. **Case**: We present a boy with infantile-onset epileptic encephalopathy, dystonia, cerebellar atrophy, and asymmetric spasticity in whom trio WES identified two heterozygous *SHQ1* variants in trans: the maternally inherited c.5T>C (p.Leu2Pro) variant and the paternally inherited c.828_831del (p.Asp277SerfsTer27) variant. Both SHQ1 variants remain classified as variants of uncertain significance (VUS) under ACMG criteria. Based on the available genetic evidence, including segregation in trans, and the close phenotypic concordance, SHQ1-related NEDDS is considered the most likely clinical diagnosis; however, the molecular findings do not independently establish the diagnosis. At 5 years and 4 months, assessment combined neurological and physiotherapeutic examination, Vojta-derived Locomotion Stages, and an adapted, descriptive item-level Bayley-4 assessment conducted outside the normative age range. The child independently expressed LS 3 creeping, brief ineffective homologous lower-limb attempts, supported kneeling with goal-directed upward reaching, and intentional tablet-based augmentative and alternative communication despite severe expressive and motor limitations. Observed task performance during the adapted Bayley-4 assessment was heterogeneous and was interpreted solely descriptively because chronological age exceeded the normative range and administration required accommodations. After adjunctive cannabidiol was introduced, prolonged nocturnal tonic seizures requiring rescue medication became less frequent and subsequently ceased, whereas focal seizures persisted during wakefulness and overall daily seizure frequency did not show a comparable reduction. Because this was a single, unblinded observation with concomitant antiseizure-medication changes and no prospectively predefined, directly comparable observation windows, no quantitative or causal treatment effect can be inferred. The available rehabilitation exposure was reconstructed retrospectively from parental report; detailed treatment progression and standardized longitudinal outcomes were not recorded prospectively. **Conclusions**: The present case broadens the functional phenotype of SHQ1-related NEDDS and shows that standardized developmental testing alone may underestimate communicative intentionality and functionally expressed abilities when motor output is severely constrained. Integrated functional assessment can refine phenotyping and identify individualized rehabilitation and assistive mobility targets. The assessment-informed physiotherapy framework is clinical and hypothesis-generating; it was not prospectively evaluated and should not be interpreted as evidence of treatment efficacy.

## 1. Introduction

The SHQ1 gene (SHQ1 homolog; H/ACA small nucleolar ribonucleoprotein assembly factor; chromosome 3p13) encodes a chaperone for H/ACA ribonucleoprotein (RNP) biogenesis. By stabilizing dyskerin (DKC1/NAP57) before complex assembly, SHQ1 supports ribosomal RNA pseudouridylation, ribosome biogenesis, spliceosomal RNA modification, and telomerase function [1,2]. Pathogenic biallelic variants can weaken this interaction and disrupt RNA/protein homeostasis [1,2,3].

Experimental SHQ1 depletion and patient-associated variants impair neuronal migration and neurite development and alter dopaminergic signaling [3], linking the molecular defect to motor and postural dysfunction. Cerebellar and white-matter abnormalities have been described in SHQ1-related disease [1,4,5,6]. The broader clinical spectrum includes hypotonia, dystonia, dysmetria or ataxia, impaired postural reactions, and corticospinal spasticity [1,4,5,6,7,8,9,10,11]; seizures can further restrict motor learning. Thus, the physiotherapy phenotype reflects central motor-network dysfunction rather than musculoskeletal disease.

To date, fewer than 20 affected individuals with SHQ1-related neurodevelopmental disorder with dystonia and seizures (NEDDS) have been reported worldwide. Although the core phenotype includes developmental delay, epilepsy, dystonia, and cerebellar abnormalities, detailed descriptions of functional motor organization, postural control, locomotor patterns, and comprehensive physiotherapeutic assessment remain scarce [1,4,5,6,7,8,9,10,11].

We expand the clinical and functional spectrum of SHQ1-related NEDDS by describing a patient with dystonia and cerebellar abnormalities, as well as clinical findings suggestive of corticospinal tract involvement, including asymmetric spasticity. We also provide a comprehensive neurological and physiotherapeutic characterization of motor function, postural organization, locomotor patterns, and communication abilities by integrating structured developmental testing with detailed functional assessment. These findings are used to formulate a clinical, hypothesis-generating physiotherapy framework that explicitly distinguishes observed assessment findings, therapeutic elements used in routine care and reported retrospectively, and recommendations and outcome measures proposed for future care and monitoring.

## 2. Case Report

This case report was prepared in accordance with the CARE (CAse REport) guidelines. The completed CARE checklist is provided as Supplementary File S1.

### 2.1. General Medical History and Genetic Evaluation

The patient, a Polish male infant, was born at term (41 weeks’ gestation) to non-consanguineous parents via spontaneous vaginal delivery following a prolonged labor. His birth weight was 3120 g, and his Apgar score was 10. The neonatal period was largely unremarkable, except for mild ventricular asymmetry noted on routine cranial ultrasonography. His early developmental milestones were reported as normal until 4.5 months of age, when he exhibited an abrupt onset of irritability, hypersomnolence, loss of social engagement, and motor regression, including loss of the ability to roll over. The parents also recalled a concurrent deterioration in head control and prone upper-limb support. No comparably marked loss of previously acquired motor abilities was reported later. At 5 months of age, the patient developed flexion spasms occurring in tight clusters (up to 60 events over 10 min) accompanied by impaired interictal consciousness. Electroencephalography (EEG) demonstrated a hypsarrhythmic pattern, establishing a clinical diagnosis of West syndrome. Initial treatment consisted of vigabatrin (150 mg/kg/day), pulsed intravenous methylprednisolone (30 mg/kg/day), and pyridoxine (25 mg/kg/day). Following a transient, partial response, the spasms recurred. The introduction of a ketogenic diet (3:1 ratio) at 6 months of age resulted in the first sustained clinical and electrographic improvement, with EEG showing normalization and the appearance of normal sleep-stage patterns. Over the subsequent 3.5 years, the patient’s epilepsy proved highly drug-resistant, remaining refractory to twelve antiseizure medication regimens used as monotherapy or polytherapy. The seizure semiology evolved into a mixed myoclonic-atonic-tonic disorder, consistent with Lennox–Gastaut syndrome (LGS). On 16 February 2024, highly purified cannabidiol (CBD; Epidyolex^®^) was introduced under a national therapeutic program at 0.3 mL once daily and subsequently titrated. At the follow-up on 26 March 2024, the dose was 0.6 mL twice daily (10.16 mg/kg/day). By August 2024, it had reached 1.1 mL twice daily (19.4 mg/kg/day). The same dose was documented in November 2024, after which an increase in the evening dose to 1.2 mL was recommended. Following cannabidiol initiation, prolonged nocturnal tonic seizures that had previously required rescue medication became less frequent and subsequently ceased; focal seizures during wakefulness persisted, and overall daily seizure frequency did not show a comparable reduction. Seizure frequency during cannabidiol treatment was documented clinically, including through a caregiver-maintained seizure diary; however, the available retrospective data did not permit reconstruction of prospectively predefined and directly comparable baseline and follow-up observation windows. Therefore, no quantitative treatment-effect estimate is presented. At cannabidiol initiation, clonazepam was replaced with clobazam at 2.5 mg twice daily; this dose remained unchanged in the available 2024 follow-up documentation. Other antiseizure treatment was modified during this period, including cannabidiol titration and subsequent tapering and discontinuation of ethosuximide. On 13 March 2025, measured concentrations of clobazam and N-desmethylclobazam (norclobazam) were within the laboratory therapeutic ranges. Increased salivation was recorded at the first follow-up after cannabidiol initiation; subsequent visits documented no adverse events. Somnolence or sedation, gastrointestinal symptoms, and reduced appetite were not documented, and follow-up liver-function tests remained within the reference ranges. New gross motor abilities were observed during the same general period, but this temporal co-occurrence does not establish causality. Additionally, at age 20 months, during a gastroenterological workup, the patient was diagnosed with hypogammaglobulinemia, characterized by immunoglobulin G (IgG) and immunoglobulin A (IgA) deficiency, which required intravenous immunoglobulin (IVIG) therapy, subsequently leading to a gradual normalization of IgG levels. IVIG had therefore been initiated before cannabidiol therapy; however, the available retrospective records did not permit precise reconstruction of the duration of IVIG treatment or its temporal overlap with cannabidiol exposure. Extensive biochemical and metabolic screening was performed to rule out metabolic disease. Tandem mass spectrometry, urinary organic acids, cerebrospinal fluid (CSF) amino acids, pterin profiles, and transferrin isoforms were all normal. A mild decrease in CSF neurotransmitter metabolites and 5-methyltetrahydrofolate was recorded, though this occurred during concurrent vigabatrin therapy. The molecular genetic workup spanned four years (Table 1). Initial karyotyping, multiplex ligation-dependent probe amplification (MLPA) for Rett syndrome, and methylation studies for Angelman syndrome were normal. A targeted next-generation sequencing (NGS) epilepsy panel (330 genes) in April 2021 was non-diagnostic, identifying only a heterozygous variant in *SGSH* (c.1322G>A), indicating carrier status for mucopolysaccharidosis type IIIA (Sanfilippo syndrome). The first whole-exome sequencing (WES) in trio version (proband and both parents) performed in May 2022 identified compound heterozygous variants of uncertain significance (VUS) in *SHMT2* (c.914G>A and c.1183C>T) and a de novo 2.7 kb 16p11.2 microdeletion involving *KAT8*. The *SHMT2* variants were considered unlikely to be causative due to the absence of cardiomyopathy, which is a hallmark feature of *SHMT2*-related neurodevelopmental disorder. Finally, trio WES reanalysis performed at an independent academic center in May 2024—post-dating the first definitive publications linking *SHQ1* to neurodevelopmental phenotypes—identified two heterozygous *SHQ1* variants in trans (NM_018130.3): the maternally inherited missense variant c.5T>C (p.Leu2Pro), mapping to the N-terminal CHORD–Sgt1 (CS) domain, and the paternally inherited frameshift variant c.828_831del (p.Asp277SerfsTer27), which introduces a premature termination codon. Repeat trio WES in 2025 confirmed both variants in trans and did not identify an alternative molecular explanation. Both SHQ1 variants remain classified as variants of uncertain significance (VUS) under ACMG criteria. The c.5T>C variant had not been previously reported, whereas c.828_831del is a recurrent allele with conflicting interpretations in ClinVar and prior reports in *SHQ1*-related phenotypes. Based on the available genetic evidence, including segregation in trans, and the close phenotypic concordance, SHQ1-related NEDDS (OMIM #619922) is considered the most likely clinical diagnosis; however, the molecular findings do not independently establish the diagnosis.

### 2.2. Brain Imaging

Brain MRI performed in April 2022 demonstrated atrophic/dysplastic changes involving the cerebellar vermis and both cerebellar hemispheres, predominantly cortical in distribution. The comparative radiology report additionally described normalization of previously increased signal intensity within the thalami, globi pallidi, brainstem, and cerebellar peduncles relative to earlier imaging. No diffusion restriction or pathological contrast enhancement was present. The ventricular system was symmetric, and a posterior fossa abnormality consistent with a Blake’s pouch cyst/Dandy–Walker continuum variant was identified.

Follow-up MRI performed in August 2023 showed that the atrophic/dysplastic changes in the cerebellar vermis and hemispheres remained stable compared with the April 2022 examination. Small, nonspecific FLAIR hyperintensities were present in the frontal and parietal white matter. Prominent perivascular spaces were also noted and were described as unchanged from the previous examination. No diffusion restriction or pathological contrast enhancement was observed. The fourth ventricle remained enlarged and open inferiorly. The posterior fossa configuration was again considered consistent with a Blake’s pouch cyst/Dandy–Walker continuum variant, without vermian elevation or the other classical features of Dandy–Walker malformation. The supratentorial ventricular system remained symmetric and undisplaced, and the subarachnoid spaces were preserved.

### 2.3. Assessment of Spontaneous Motor Activity, Reflex Responses, and Postural Control

The functional assessment was conducted on 22 January 2026, when the patient was 5 years and 4 months old.

Motor performance was additionally classified using Vojta-derived Locomotion Stages (LS), as operationalized by Sanz Mengibar et al. [12] (Table 2). LS was assigned according to the highest independently and functionally expressed locomotor pattern. The patient generated purposeful forward progression primarily through upper-limb support and propulsion, whereas the lower limbs did not provide effective propulsion, corresponding to LS 3 (creeping). Intermittent attempts containing an LS 4-like homologous pattern (“bunny hopping”) were observed when he approached a self-selected target; however, these attempts were short-lived and did not produce effective forward progression. Accordingly, the patient’s highest independently and functionally expressed locomotor pattern was classified as LS 3. Functionally, he remained dependent for sitting and transfers and, based on parental report, had not yet received training in independent wheeled mobility, despite purposeful self-initiated floor locomotion. Given his goal-directed behavior and high intrinsic motivation, the family was considering an assessment for powered mobility operated using an individually selected control interface, initially a joystick.

According to retrospective caregiver recall, rolling re-emerged at approximately 2 years of age, purposeful communication and use of augmentative and alternative communication began at approximately 2 years, and independent floor mobility through creeping emerged at approximately 4 years. Quadruped positioning, supported kneeling, and pulling to stand or standing with assistance were reported at approximately 5 years. These ages are approximate and were not established through serial standardized assessments. Their temporal relationship with changes in medication or individual rehabilitation methods cannot be determined.

Without parental support, the patient is unable to assume an upright sitting position. When passively placed in sitting, he cannot maintain the position independently and requires support at the lumbar and thoracic spine. In sitting, he demonstrates pronounced thoracic kyphosis with lateral trunk flexion, more frequently to the right. Occasional spontaneous attempts at sitting result in a posture resembling side sitting. During locomotion, alternating movements of the upper limbs are observed, accompanied by homologous activity of the lower limbs and a lack of active knee loading during hip extension. During attempts to assume a quadruped position and during hopping, the hips remain flexed with a tendency toward internal rotation; the right lower limb is more active, while the left remains stiffer. The patient flexes the toes and forefoot of the left foot, positioning it in more pronounced plantar flexion. Both lower limbs demonstrate internal rotation at the hip joints. Despite high motivation to climb, limited knee weight-bearing together with cerebellar signs, dysmetria, dystonia, and central hypotonia prevented attainment of supported standing. He was nevertheless able to attain a supported kneeling position and reach upward toward a self-selected book with one upper limb while maintaining contralateral upper-limb support (Figure 1C). This demonstrated emerging antigravity postural control but did not meet the criteria for independent sitting.

### 2.4. Manual Function and Communication

The patient demonstrates high motivation for both locomotion and communication. The patient is non-verbal and communicates using a tablet-based augmentative and alternative communication (AAC) system; he can introduce himself and indicate the wish to terminate an activity (Figure 2). He does not yet communicate physiological needs. Communication is most effective in the prone position with weight-bearing through the elbows. Phasic activity of the right upper limb predominates, while the left upper limb is used to a limited extent. Goal-directed movements show marked dysmetria and compensatory strategies consistent with cerebellar dysfunction. Coexisting dystonia and dysmetria substantially limit accurate reaching toward the communication device. Left-predominant spasticity, including adduction of the thumb and first metacarpal, further impairs precision grip, climbing, and upper-limb weight-bearing.

### 2.5. Postural and Reflex Responses

Protective reactions are rudimentary, delayed, and limited to the right upper limb (parachute reaction), resulting in falls during attempts at locomotion in the quadruped position (Figure 3A). Axial stability is limited. During the traction response, the patient activates head flexion and, with considerable effort, maintains cervical muscle activity until reaching the sitting position (Figure 3B).

In the axillary suspension response, extension of the lower limbs is observed (left > right), along with plantar flexion of the feet, hip adduction, and knee extension. The upper limbs remain adducted with clenched fists; the head is maintained in the sagittal plane (Figure 3C). Responses during Vojta suspension were analogous, including rigid extension of the lower limbs (left > right) and lateral trunk flexion toward the dependent side, regardless of the direction in which the patient was tilted (Figure 3D). Due to increasing emotional distress, further testing was discontinued.

### 2.6. Neurological and Functional Findings

The clinical presentation indicates a coexistence of spasticity and dystonia. A positive Babinski sign, more pronounced on the left side, and velocity-dependent increase in muscle tone (rapid, rigid first resistance during attempted ankle dorsiflexion) support the presence of a spastic component, more evident on the left. Exaggerated tendon reflexes were noted in the biceps brachii and patellar tendon. Dystonic symptoms intensified in response to auditory, tactile, and emotional stimuli. During the assessment, brief epileptic episodes of a myoclonic nature were observed.

During tablet-based communication, hypermetric saccadic eye movements with frequent overshooting of the target and associated head movements were evident, with saccades more pronounced in the frontal plane. Attempts to stabilize the head increased anxiety, limiting the objectivity of saccadic precision assessment under externally supported postural conditions.

### 2.7. Developmental and Caregiver-Reported Functional Assessment

A developmental assessment was performed on 7 February 2026, when the patient was 5 years and 4 months old, using the Bayley Scales of Infant and Toddler Development, Fourth Edition (Bayley-4) [13]. The assessment was administered by a certified therapist. Bayley-4 was not used as a rehabilitation outcome measure but as a structured, item-level framework permitting concurrent description of cognitive, receptive and expressive communication, and fine and gross motor functioning. This approach was selected because severe motor and expressive impairments limited the valid administration of developmental measures appropriate for the patient’s chronological age. To facilitate task access, the patient remained seated with postural support in his own seating device (an adapted chair or non-powered wheelchair; the available retrospective information did not distinguish between these), used his AAC tablet, was allowed extended response time and several attempts when needed, and completed the assessment in the presence and with the support of both parents. The total assessment time was extended, and breaks were provided. These administration details were obtained from the certified therapist who conducted the assessment. Because these accommodations differed from standardized administration, they further preclude normative interpretation. In contrast, the Gross Motor Function Measure-88 (GMFM-88) addresses the narrower domain of gross motor capacity and does not capture the cross-domain phenotypic profile examined in this case; it was therefore considered complementary rather than a substitute for Bayley-4 [14]. The absence of concurrent GMFM-88 and Segmental Assessment of Trunk Control (SATCo) assessments is acknowledged as an important methodological limitation, and GMFM-88, SATCo, and prospectively defined Goal Attainment Scaling (GAS) are proposed for future serial monitoring [14,15,16]. The Bayley-4 technical manual specifies an upper normative age limit of 42 months [13]; therefore, because the patient’s chronological age was 64 months, no age-normed composite scores or developmental-age equivalents were calculated. Raw scores are retained solely to document descriptive item-level task access and must not be interpreted as validated developmental ages. Marked intra-individual variability was observed: the patient completed selected later items while not completing some earlier items, consistent with severe motor and expressive-output constraints and a disharmonious functional profile. The cognitive raw score was 41; completed tasks clustered predominantly in the earlier portion of the item sequence, with isolated success on later items. Receptive communication yielded a raw score of 25 and showed a similarly non-sequential pattern across the item sequence. Expressive communication yielded a raw score of 12; performance was substantially constrained by the patient’s non-verbal status and reliance on AAC. Fine motor performance (raw score 18) was limited by dystonia, dysmetria, spasticity, and reduced precision of goal-directed hand use. Gross motor performance yielded a raw score of 50 and was relatively stronger, although expressed through pathological postural and locomotor patterns. Overall, the findings describe markedly heterogeneous task access, with relatively stronger receptive and gross motor performance than expressive and fine motor output. They should not be interpreted in isolation as a measure of global cognitive ability, learning potential, or developmental age.

At 5 years and 11 months of age (8 September 2026), the patient’s parents completed the online English-language Content-Balanced version of the Pediatric Evaluation of Disability Inventory Computer Adaptive Test (PEDI-CAT) [17]. Both parents were fluent in English, and no translation or interviewer assistance was used. The assessment characterized the patient’s typical current functional performance across the Daily Activities, Mobility, Social/Cognitive, and Responsibility domains. No walking device was reported, and the patient used a manual wheelchair that he did not self-propel. Domain scaled scores (standard errors) were 45 (0.84) for Daily Activities, 53 (1.66) for Mobility, 54 (1.02) for Social/Cognitive, and 37 (1.98) for Responsibility. The corresponding age-normed T-scores and percentiles, generated using the PEDI-CAT Content-Balanced assessment on Pearson’s Q-global platform (report dated 8 September 2026, bearing the designation “1.1”), were 15/<1st percentile, <10/<1st percentile, 13/<1st percentile, and 28/2nd percentile, respectively.

Caregiver responses indicated marked dependence in self-care and daily activities, severe limitations in upright mobility and unsupported sitting, and predominant caregiver responsibility for complex daily tasks, while selected basic floor-level mobility and intentional communication behaviors were reported. The PEDI-CAT was completed in English using US normative data, without a validated Polish cross-cultural adaptation. Although both parents were fluent in English, the scores should therefore be interpreted cautiously and primarily as descriptive information about the patient’s everyday functional performance. PEDI-CAT is a caregiver-reported measure of typical activity performance rather than a direct assessment of motor capacity or postural control. This single later assessment was not used to infer longitudinal change or treatment efficacy.

### 2.8. Retrospectively Reported Rehabilitation Exposure and a Hypothesis-Generating Physiotherapy Framework

Physical therapy was provided as part of the patient’s routine clinical care. The authors did not provide this treatment; their involvement was limited to the diagnostic and functional assessment visits described in this report. Accordingly, the manuscript distinguishes (i) findings observed during these assessment visits, (ii) therapeutic elements used in routine care and reported retrospectively by the parents, and (iii) recommendations and outcome measures proposed for future care and monitoring.

According to caregiver report obtained during manuscript revision, physical therapy began at approximately 6 months of age. During the preceding 12 months, it was generally provided two to three times per week, with each session lasting approximately 45 min; earlier frequency and session duration were described as broadly similar. The therapeutic focus was adjusted over time, from facilitation of head control to upper- and lower-limb weight-bearing and supported verticalization. Within this overall frequency, the current schedule comprised sessions combining elements of NDT/Bobath with Cuevas Medek Exercises (CME), and one separate weekly session conducted according to the Vojta therapy concept. Functional practice and selected PNF techniques were incorporated where appropriate. Structured therapeutic exercises were performed at home infrequently; caregivers instead encouraged spontaneous motor activity during play. These data were reconstructed retrospectively and were not supported by contemporaneous treatment logs or standardized longitudinal outcome measurements.

Speech and language therapy reportedly began at approximately 1 year of age and, during the preceding 12 months, was generally provided once weekly for approximately 45–60 min. Its initial focus was feeding difficulties, followed by augmentative and alternative communication, vocalization, and orofacial myofunctional therapy. A tablet-based AAC system was introduced by a speech and language therapist at approximately 2 years of age and was subsequently used daily across home, therapy, educational, and community settings. The caregivers also reported weekly AAC-focused sessions and regular or intermittent hand therapy, sensory-integration activities, vision therapy, and feeding or swallowing support. The frequency and duration of these additional interventions were not consistently documented.

The Vojta-derived Locomotion Stages (LS) were used to record the patient’s highest spontaneously and independently expressed locomotor pattern. For treatment planning, the classification was not interpreted in isolation but together with observations of spontaneous activity, postural reactions, dystonic responses, spasticity, lower-limb alignment, and the quality of weight-bearing. This integrated interpretation related the identified neuromotor impairments to their functional consequences for locomotion and to specific therapeutic priorities.

The integrated assessment was used to organize the therapeutic content reported by the parents into a clinical, hypothesis-generating framework. According to parental report, the elements used in routine care included active task-specific practice, positioning, graded weight-bearing, therapist-guided facilitation, environmental adaptation, caregiver involvement, and assistive technology for AAC access. The emphasis on active task-specific practice is consistent with evidence from pediatric neurorehabilitation [18,19], while graded weight-bearing, selected NDT/Bobath and PNF elements, and elements of Vojta therapy were individualized according to each functional objective. These elements were not delivered as a single standardized, method-specific protocol. Their use is described retrospectively and should not be interpreted as evidence of effectiveness.

The patient had received an active manual wheelchair several months before the caregiver report. Only a few attempts at self-propulsion had been undertaken, and no structured wheelchair-mobility training was reported. No powered-wheelchair trial had been performed. The patient also used an adaptive seating system several times daily, a stander approximately once weekly, and orthoses. Future optimization of manual wheelchair use and evaluation of patient-controlled powered mobility were included as recommendations intended to broaden independent exploration, environmental interaction, and participation, rather than as interventions already provided.

Contemporaneous treatment logs were not available. The therapeutic elements listed in Table 3 were used in routine care according to parental report, but treatment progression, objective adherence, and outcomes were not recorded prospectively. The standardized tests and scales in the final column were not administered and are proposed solely for future monitoring. Table 3 therefore documents reported treatment content but does not provide evidence of treatment efficacy.

Prospective monitoring should use a standardized starting position, predefined assistance levels, and comparable task conditions. GAS goals should be established before treatment rather than scored retrospectively. Safety and tolerability monitoring should include pain, fatigue, distress, increased dystonia, and seizure-related events. Postural support should also be standardized during AAC tasks, because the level of trunk support can directly influence reaching performance [20].

### 2.9. Clinical Timeline

The clinical, treatment, rehabilitation, and functional chronology is summarized in Table 4.

## 3. Discussion

SHQ1 acts as an early assembly factor for H/ACA ribonucleoproteins, binding dyskerin before the recruitment of NAF1 and other components of the mature complex [1,2]. Biallelic SHQ1 variants have been associated with a broad neurological continuum, ranging from severe neurodevelopmental disorder with dystonia and seizures (NEDDS) to predominantly dystonic phenotypes with relatively preserved cognition and motor function [1,4,5,6,7,8,9,10,11]. This marked phenotypic variability remains insufficiently explained, and a consistent genotype–phenotype relationship has not yet been established.

In the present patient, a detailed functional physiotherapeutic assessment performed at 5 years and 4 months refined the clinical phenotype beyond the information provided by the genetic findings and structured developmental testing alone. The examination combined observation of self-initiated locomotion and goal-directed behavior with assessment of axial and sitting control, upper- and lower-limb weight-bearing, movement symmetry, protective and postural reactions, muscle tone, goal-directed reaching, manual access to an augmentative and alternative communication device, and responses to sensory and emotional stimuli. This approach revealed clinically relevant motor organization, preserved intentionality and therapeutic priorities that could not be inferred from the genetic findings alone.

The adapted Bayley-4 assessment and the functional-neurological examination provided complementary information. Item-level performance showed restricted and disharmonious access to developmental tasks, particularly in the fine motor and expressive communication domains, alongside access to selected later items. Because the patient’s chronological age of 64 months exceeded the instrument’s upper normative age limit of 42 months, all raw and item-level findings are descriptive only and are neither age-standardized scores nor developmental-age estimates [13]. Supported seating, AAC use, extended response time, several attempts, parental presence and support, and breaks may all have influenced task performance. Severe dystonia, dysmetria, spasticity, impaired postural stabilization, and imprecise reaching provided additional constraints. Accordingly, the Bayley-4 findings should be interpreted only as observed task access under these adapted conditions and not as an isolated measure of global cognitive ability or learning potential.

The subsequent parent-reported PEDI-CAT profile added an age-range-appropriate description of typical real-world functional performance and was broadly consistent with the observed severe dependence, preserved selected floor-level mobility, and communicative intentionality [17]. The PEDI-CAT was completed on 8 September 2026, approximately seven months after the Bayley-4 assessment on 7 February 2026 and more than seven months after the neurological and physiotherapeutic assessment on 22 January 2026. It is not capacity-based and does not directly quantify postural control. It therefore complements, but does not validate, the adapted Bayley-4 findings or replace the missing concurrent GMFM-88 and SATCo assessment. Interpretation is further limited by single-time-point parental proxy reporting, use of United States normative data, and lack of a validated Polish-language cross-cultural adaptation.

No prospectively standardized longitudinal motor or postural outcome measurements were available. The January neurological and physiotherapeutic assessment, February Bayley-4 assessment, September PEDI-CAT, and retrospectively reconstructed rehabilitation history therefore cannot establish treatment-related change. The proposed physiotherapy framework should be regarded as hypothesis-generating rather than as evidence of treatment efficacy. GMFM-88 would have provided a more suitable framework for repeated measurement of gross motor change, and SATCo would have provided a structured assessment of segmental trunk control [14,15]. These measures would not, however, have addressed the multidomain phenotypic question that motivated the descriptive use of Bayley-4, because they do not assess cognition, receptive or expressive communication, and fine motor function. They should therefore be regarded as complementary rather than substitutive. Nevertheless, Bayley-4 cannot serve as an objective longitudinal outcome measure in this patient: age-standardized change scores are unavailable, and raw or item-level changes may reflect variations in postural support, motor access, fatigue, task familiarity, or the non-standardized accommodations rather than rehabilitation-related change. Future progress should therefore be tracked using GMFM-88, SATCo, and prospectively defined GAS under standardized task conditions [14,15,16].

This interpretation is consistent with recent evidence showing that different developmental and neurological assessment tools capture related but non-interchangeable aspects of child development. Deng et al. reported a strong correlation between raw scores on the Bayley-4 gross motor subtest and the motor-focused Alberta Infant Motor Scale, but only fair categorical agreement between Bayley-4 and the Hammersmith Infant Neurological Examination in children assessed at approximately 12 months of age [21]. Although these findings were obtained in a younger population and do not validate the use of Bayley-4 beyond its normative age range, they support the principle that standardized developmental scores and detailed neurological examination provide complementary information. In our patient, purposeful use of tablet-based augmentative and alternative communication, sustained motivation, task-directed behavior, and the ability to communicate self-identification and termination of an activity indicated communicative intentionality and task engagement that were not adequately represented by expressive language and fine motor scores alone. This highlights the importance of integrating standardized developmental testing with detailed functional analysis in order to accurately interpret developmental level and to guide therapeutic planning. Comparable principles have been emphasized in other ultra-rare genetic neurodevelopmental disorders, where detailed and standardized characterization of motor, cognitive, and adaptive functioning is considered essential for phenotypic delineation and for planning individualized, multidisciplinary care, including physical and occupational therapy [22].

Spontaneous mobility was additionally classified using Vojta-derived Locomotion Stages (LS), as operationalized by Sanz Mengibar et al. [12]; this classification should not be understood as a form of Vojta therapy. The LS levels constitute an ordered functional classification of postural-locomotor capacity. In 462 children with cerebral palsy, LS showed high reliability and was strongly associated with gross motor environmental performance [12]. This ordering reflects differences in the complexity of postural control and independent locomotor function observed at assessment rather than a necessary sequence of individually expressed locomotor patterns. LS 4 describes a pathological homologous crawling pattern (“bunny hopping”) and has no physiological equivalent in typical motor development. In the present patient, the highest effective locomotor pattern remained LS 3. Brief homologous lower-limb attempts showed additional lower-limb recruitment but did not generate effective forward progression or alter the classification.

Beyond assigning a categorical level, LS added an integrative link between automatic postural control observed during postural reactions and spontaneous activity, the impairments identified on neurological examination, and their consequences for purposeful locomotion. The same functional limitations—including asymmetric support-extension capacity, spasticity, dystonia, restricted lower-limb dissociation, and insufficient active knee loading—were evident during effective creeping and the brief homologous attempts. Therapeutic priorities were therefore derived from these component impairments rather than from pursuit of a higher LS category. They included improving alternating weight transfer, lower-limb dissociation, active knee support, and progression of the pelvis over a stabilized femur toward hip extension. Improvement in these capacities may facilitate more reciprocal locomotion and the expression of motor abilities in higher antigravity positions. Because the measurement properties and functional associations of LS were established primarily in children with cerebral palsy, its application in SHQ1-related NEDDS remains exploratory functional phenotyping rather than a disease-specific validated outcome or prognostic framework.

According to parental report, therapeutic activities aimed at reducing reliance on the homologous pattern included facilitation of alternating weight-bearing and reciprocal limb activity, selected positions used in Vojta therapy, such as Position I, active task-specific activities, and play incorporating NDT/Bobath handling and facilitation strategies. The rationale for these activities represents a working therapeutic hypothesis derived from the integrated assessment rather than a demonstrated treatment effect. Treatment dose, progression, and repeated standardized outcomes were not recorded prospectively; therefore, the present case cannot establish that therapy modified the patient’s spontaneous locomotor pattern or that any single intervention component improved function. The monitoring framework proposed in Section 2.8 is intended to support future documentation using reproducible task conditions, predefined assistance levels, and prospectively selected goals.

Within routine care, NDT-derived handling was reported as being used only as an adjunct to active, goal-directed, and task-specific practice. In children with cerebral palsy or at high risk of cerebral palsy, a meta-analysis found no difference in motor function between NDT and control conditions, whereas the pooled comparison favored explicitly activity-based approaches, although heterogeneity was considerable [23]. For Vojta therapy, a small randomized controlled trial involving 20 children with central hypotonia reported improvements in selected postural measures and a greater change in total GMFM-88 scores compared with general physical therapy after six weeks [24]. However, the small, heterogeneous sample and short intervention period limit the generalizability of these findings, which do not establish efficacy in SHQ1-related NEDDS. The present case cannot determine the contribution of either approach to the patient’s functional abilities. Therefore, elements derived from these approaches are described here as individualized adjuncts within an active functional program rather than as independently validated, diagnosis-specific protocols.

This framework also links physical therapy with communicative autonomy. In supported prone positioning, elbow or forearm weight-bearing appeared to stabilize the trunk and shoulder girdle while freeing the contralateral upper limb for tablet access. We hypothesize that improving active segmental trunk control and unilateral upper-limb weight-bearing may reduce the need for external support and facilitate more precise contralateral reaching toward the AAC device. However, because AAC selection accuracy and response latency were not measured prospectively, this remains a clinical hypothesis rather than a demonstrated treatment effect. Evidence that passive external trunk support influences seated posture and reaching in children with cerebral palsy provides indirect biomechanical support for this hypothesis and justifies standardizing postural support during repeated AAC assessments [20]. Future evaluation of powered mobility should aim to increase independence and participation alongside continued practice of active transitions and safe, supported upright activities.

Published descriptions of motor function in SHQ1-related disease are sparse and predominantly qualitative. The most severely affected individuals were non-ambulatory or wheelchair-dependent and had profound developmental delay, hypotonia, absent speech, and complex hyperkinetic movements, including dystonia, chorea, athetosis, and ballism [1,10]. In the pediatric cohort reported by Chi et al., profound hypotonia and paroxysmal dystonia were the leading early manifestations [5]. A subsequent comparative synthesis indicated that six of eight patients were non-ambulatory, one walked with assistance, and one had an ataxic gait [6]. Conversely, AlHargan et al. described a patient who retained supported walking despite lower-limb spasticity and ataxia, whereas his affected sister was wheelchair-dependent [4]. Indelicato et al. reported delayed motor milestones followed by a mixed hyperkinetic disorder combining dystonia, chorea, myoclonus, and truncal hypotonia [7]. More recently, Ly and Lee described early impaired cervical motor control, hypotonia, choreiform movements, and oromotor hypotonia, but also emphasized continued functional developmental gains [8]. At the mildest end of the broader SHQ1-related spectrum, an individual reported by Revert Barberà et al. retained independent walking and running, with only mild limb and cervical dystonia and difficulty during tandem gait [9].

Against this background, the purposeful, self-initiated mobility demonstrated by our patient suggests relative preservation of functional motor initiative compared with the severe end of the NEDDS spectrum. Although pathological creeping and intermittent hopping should not be equated with independent bipedal walking, these patterns enabled goal-directed movement within the environment. To our knowledge, homologous hopping has not been specifically documented in peer-reviewed SHQ1 reports [1,4,5,6,7,8,9,10,11] and may represent an individualized compensatory strategy in response to combined postural instability, cerebellar dysmetria, dystonia, spasticity, and limited knee loading. Previous SHQ1 reports have generally classified affected individuals as averbal or as having speech delay without detailed assessment of functional non-verbal communication [1,4,5,6,7,8,9,10,11]. Effective tablet-assisted communication therefore constitutes an important functional feature and demonstrates preserved communicative intentionality despite severe expressive speech and motor impairment.

In the present patient, new gross motor abilities were observed during the same general period as improved control of prolonged nocturnal tonic seizures. Highly purified cannabidiol (CBD; Epidyolex^®^) is an established adjunctive treatment for seizures associated with Lennox–Gastaut syndrome. In the randomized phase III GWPCARE3 and GWPCARE4 trials, CBD at 10–20 mg/kg/day significantly reduced drop-seizure frequency compared with placebo, with generally manageable but more frequent adverse events [25,26]; long-term open-label follow-up showed sustained reductions in drop and total seizures for up to 156 weeks [27]. To our knowledge, highly purified CBD has not previously been described in SHQ1-related NEDDS [1,4,5,6,7,8,9,10,11]. In our patient, adjunctive cannabidiol was temporally followed by a reduction in prolonged nocturnal tonic seizure burden and by the subsequent resolution of these seizures, whereas focal seizures persisted during wakefulness. Seizure frequency was documented clinically, including through a caregiver-maintained diary, but prospectively predefined and directly comparable observation windows were unavailable. Treatment was unblinded, cannabidiol initiation coincided with replacement of clonazepam by clobazam, and cannabidiol titration and ethosuximide tapering occurred during follow-up; therefore, no quantitative or causal treatment effect can be established. The motor changes observed during the same period may alternatively reflect natural developmental maturation, ongoing rehabilitation, concomitant clobazam treatment, and the broader clinical course, including possible overlap with IVIG therapy. Observer and reporting bias cannot be excluded. Accordingly, the seizure and motor findings should be interpreted as temporally associated clinical observations only.

Both *SHQ1* variants remain classified as variants of uncertain significance under ACMG criteria; genotype–phenotype inferences therefore require caution and should be interpreted together with segregation and clinical concordance. The recurrent c.828_831del (p.Asp277SerfsTer27) allele identified in our patient has previously been reported in association with both severe neurodevelopmental presentations and milder, predominantly dystonic phenotypes [6,7,8,9,10]. Protein-level nomenclature varies across transcript versions: Ly and Lee [8] reported the same c.828_831del frameshift as p.Glu276fs*12 using NM_018130.4. In contrast, to our knowledge, the maternally inherited c.5T>C (p.Leu2Pro) variant has not been reported in the peer-reviewed *SHQ1* literature [1,4,5,6,7,8,9,10,11]. Its localization within the N-terminal CS domain is biologically relevant, given the role of this domain in interactions required for H/ACA ribonucleoprotein assembly [1,2]; nevertheless, its individual contribution to clinical severity cannot be inferred without direct functional evidence. The coexistence of the recurrent frameshift allele with a previously unreported missense variant may contribute to the patient’s distinctive, intermediate functional phenotype, although this remains hypothetical.

Serial MRI findings in our patient are partly consistent with the evolving neuroradiological spectrum of SHQ1-related disease. Initially normal imaging followed by the emergence of cerebellar atrophy was reported by Sleiman et al., while Chi et al. observed an increase in the number of patients with cerebellar atrophy on follow-up imaging [5,10]. Cerebellar hypoplasia or atrophy, cortical atrophy, hypomyelination, and thinning of the corpus callosum have also been documented in more severely affected individuals [1,4,6]. More recently, AlBathi et al. reported diffuse symmetric hypomyelination involving the subcortical U-fibers together with mild supratentorial and cerebellar atrophy [11]. Although this white-matter distribution partially overlaps with the U-fiber abnormalities observed in our patient, the small and nonspecific lesions in our case should not be equated with diffuse hypomyelinating leukodystrophy. Accordingly, the persistent cerebellar abnormalities, which remained stable between April 2022 and August 2023, are compatible with the recognized SHQ1-associated imaging spectrum and provide a plausible structural correlate for dysmetria, axial instability, impaired postural reactions, and inaccurate goal-directed reaching.

The comparative MRI report from April 2022 described normalization of previously increased signal intensity involving the thalami, globi pallidi, brainstem, and cerebellar peduncles. Such partially regressive signal abnormalities have not been recurrently documented in the available SHQ1-related series [1,4,5,6,10,11] and should therefore be interpreted cautiously. Similarly, a Blake’s pouch cyst or Dandy–Walker continuum morphology has not been established as a characteristic feature of SHQ1-related disease and may represent an individual or incidental neuroradiological finding. Overall, integrating structured developmental assessment with analysis of spontaneous locomotion, neurological examination, and functional communication refined phenotypic description and identified specific therapeutic targets, supporting multidimensional assessment in SHQ1-related NEDDS.

## 4. Conclusions

This case expands the functional phenotype of SHQ1-related NEDDS and shows how multidomain assessment may be used to define individualized goals addressing asymmetric loading, axial control, supported kneeling, AAC access, and alternative mobility. The assessment-informed physiotherapy framework presented here is a clinical, hypothesis-generating synthesis of observed findings, retrospectively reported therapeutic content used in routine care, and recommendations for future care. It was not prospectively evaluated and does not establish treatment efficacy. Future longitudinal studies should test the proposed strategies and outcome measures under standardized conditions.

## Figures and Tables

**Figure 1:**
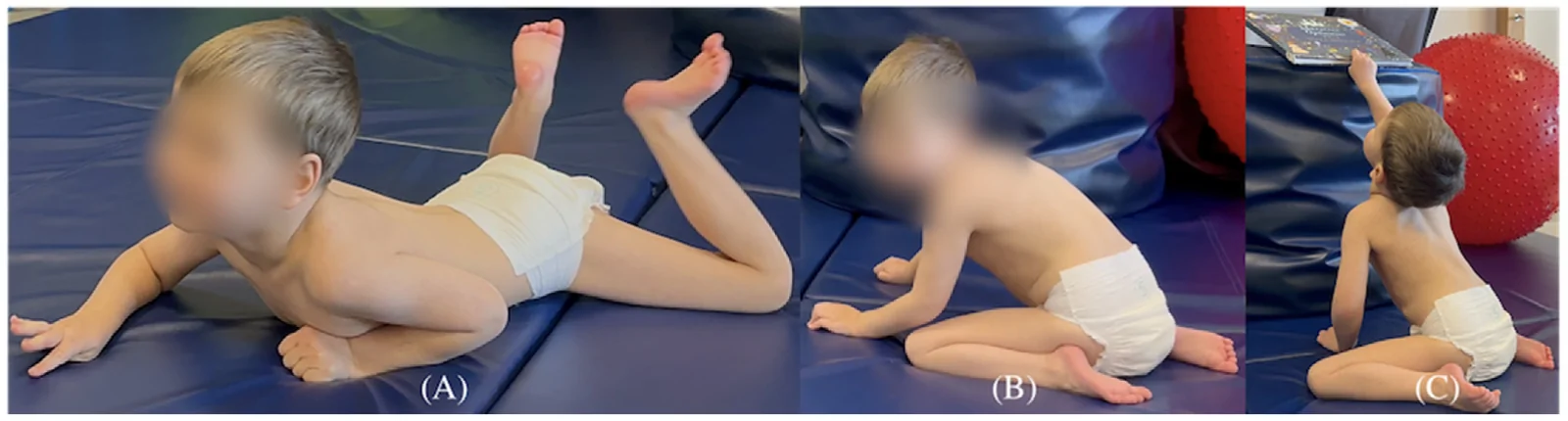
Spontaneous motor activity interpreted using Vojta-derived Locomotion Stages (LS). (**A**) Independently expressed creeping pattern corresponding to LS 3. (**B**) Intermittent, ineffective homologous lower-limb attempts resembling the LS 4 pattern; these did not generate effective independent locomotion. (**C**) Supported kneeling with goal-directed upward reaching while maintaining contralateral upper-limb support.

**Figure 2:**
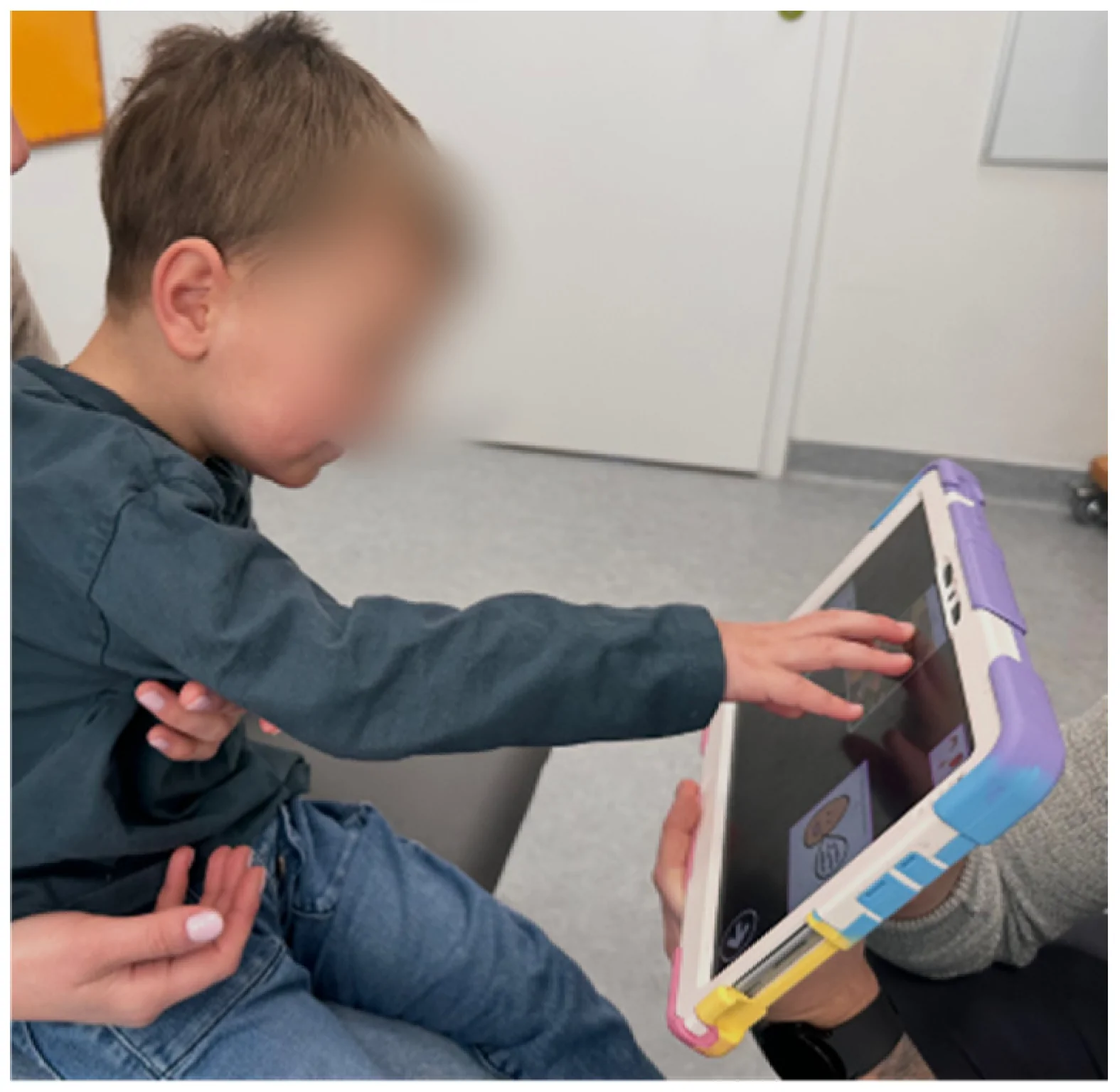
Tablet-based AAC. The patient demonstrates intentional communication, including self-introduction and the ability to express the need to terminate an activity.

**Figure 3:**
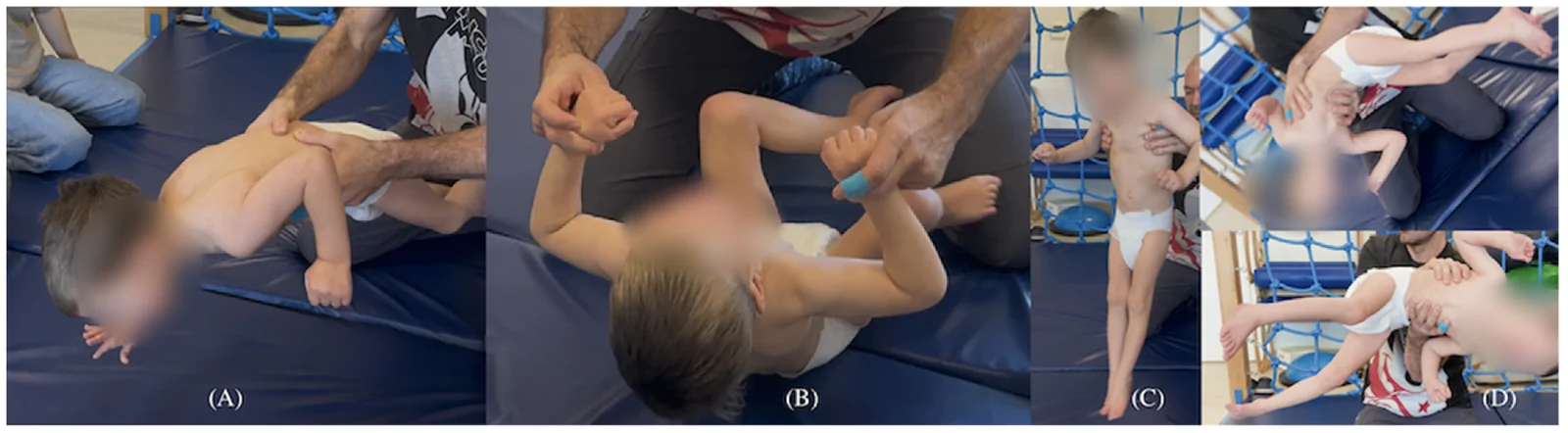
Assessment of postural responses in selected tests and suspension positions: (**A**) delayed and asymmetrical protective responses (parachute suspension); (**B**) traction response demonstrating effortful maintenance of cervical control; (**C**) axillary suspension revealing impaired postural control, with lower-limb extension (left > right), plantar flexion, hip adduction, and clenched fists; and (**D**) Vojta suspension showing a similar pattern of disrupted postural responses, including rigid lower-limb extension (left > right) and lateral trunk flexion toward the dependent side.

**Table 1:** Summary of key genetic investigations.

Test	Date	Result	Conclusion
NGS Epilepsy Panel	April 2021	*SGSH* c.1322G>A (het)	Carrier (Sanfilippo IIIA); non-causative
Trio WES (initial analysis)	May 2022	SHMT2 c.914G>A + c.1183C>T; de novo 16p11.2 del (KAT8)	VUS; plausible candidates, but phenotype atypical
MLPA Panel (Rett)	July 2022	Normal	Excluded
Trio WES (reanalysis; repeat testing)	May 2024; November 2025	*SHQ1* c.5T>C (mat) + c.828_831del (pat), confirmed in trans; no alternative explanatory finding	Both variants classified as VUS; clinical genetic evaluation considered the phenotype consistent with NEDDS

Abbreviations: het, heterozygous; del, deletion; mat, maternally inherited; pat, paternally inherited.

**Table 2:** Vojta-derived Locomotion Stages (LS) and corresponding patterns in typical motor ontogeny, based on the operational definitions reported by Sanz Mengibar et al. [12].

Stage	Operational Definition of the Locomotor Pattern	Corresponding Pattern in Typical Motor Ontogeny
0	Orientation: no contact by turning, reaching, or grasping	Holokinetic movement
1	Turning head and body toward a stimulus and reaching toward a stimulus with at least one hand	Reaching toward a toy (~4 months)
2	Onset of pathological support and grasping in the prone position	Prone forearm support with unilateral weight shift and grasp (4–5 months)
3	Creeping on one or both forearms; the legs do not generate propulsion	Creeping/commando crawling (~7 months)
4	Homologous crawling (“bunny hopping”); both legs advance simultaneously	No physiological equivalent
5	Reciprocal crawling on hands and knees in a crossed alternating pattern	Reciprocal crawling (~9 months)
6	Independent pull-to-stand and sideways cruising while holding furniture	Sideways cruising along furniture (12–13 months)
7	Independent walking, stopping, and turning without support	Walking, stopping, and turning without support (~15 months)
8	Single-leg stance for at least 3 s on one preferred side	Single-leg stance on one side (~3 years)
9	Single-leg stance for at least 3 s on both sides	Single-leg stance on both sides (~4 years)

LS is assigned according to the highest independently and functionally expressed locomotor pattern.

**Table 3:** Assessment-informed physiotherapy framework linking observed findings to functional goals, therapeutic elements reported as used in routine care, and outcome measures proposed for future monitoring. The measures in the final column were not administered in this case; no treatment effect is inferred.

Assessment Finding	Functional Goal	Therapeutic Elements Reported as Used in Routine Care	Outcome Measures Proposed for Future Monitoring (Not Administered)
Asymmetric muscle tone and dystonic posturing	Improve midline orientation and achieve more symmetrical loading	Therapist-guided positioning and controlled lateral weight shifts on a therapy ball and a suspended platform; cross-legged sitting with graded perturbations; active bimanual tasks; and graded loading through large joint surfaces to increase proprioceptive input. Manual assistance was adjusted to permit active maintenance or recovery of alignment.	Standardized video observation; individualized Goal Attainment Scaling (GAS); duration and tolerance of the position; level of assistance; frequency of loss of midline alignment.
Reduced and asymmetric knee loading with a flexion-dominant lower-limb pattern	Develop supported quadruped and kneeling and increase antigravity lower-limb activity	Assisted quadruped and supported kneeling; graded knee loading; transitions from sitting to kneeling; supported standing; and progressive reduction in pelvic or trunk assistance. Selected Vojta therapy elements, such as Position I (the first position), were used as preparatory facilitation before active task practice.	Time maintaining quadruped or kneeling; assistance level; number of successful trials; relevant lying/rolling, crawling/kneeling, and standing items from the GMFM-88 [14].
Limited axial postural control	Improve head and trunk control required for support, reaching, and AAC access	Graded external trunk support in sitting and quadruped; therapist-controlled perturbations and diagonal weight shifts, with emphasis on loading the right upper and lower limbs when clinically indicated; PNF stabilizing reversals in quadruped; resisted trunk rotation in low positions; and task-specific reaching with the minimum support required for successful performance.	Segmental Assessment of Trunk Control (SATCo) [15]; duration of head control; lowest effective level of external trunk support; number and proportion of successful reaching attempts.
Limited upper-limb weight-bearing capacity	Use one upper limb for stable support while freeing the other for reaching and AAC operation; contribute to supported upright activity	Bilateral and unilateral forearm or hand support; approximation through the shoulder girdle in side sitting while the opposite hand performed a manipulative task; resisted PNF head patterns during forearm support; alternating support and reach at a therapy table; AAC practice with therapist-adjusted postural support; and bilateral grasp of ropes, straps, or bars for grasp activation and assisted upright positioning using unstable supports.	Duration of unilateral support; AAC symbol-selection accuracy and response latency; unintended activations; assistance required; comparison of AAC performance across standardized support conditions.
Absence of independent functional mobility	Increase participation in transitions and provide safe alternative mobility	Active rolling, including resisted rolling, and transitions to side sitting; task-oriented transitions among sitting, kneeling, and supported standing, including assisted standing at a therapy table with therapist-guided pelvic control; use of a standing frame; and participation-oriented practice during daily and peer-group activities at the rehabilitation center.	Relevant GMFM-88 items [14]; prospectively defined GAS goals [16]; level of assistance during transfers; access to and participation in daily activities; caregiver-reported feasibility and burden.

**Table 4:** Chronological summary of the clinical course, treatment, rehabilitation exposure, and functional development.

Age or Date	Clinical Course and Treatment	Rehabilitation and Functional Development	Source of Information
4.5–5 months	Abrupt developmental regression at 4.5 months was followed by flexion spasms with hypsarrhythmia at 5 months; West syndrome was diagnosed.	Loss of rolling and deterioration in head control and prone upper-limb support.	Medical records and parental history
Approximately 6 months	A ketogenic diet was introduced and was followed by sustained clinical and EEG improvement.	Physical therapy began.	Medical records and parental rehabilitation history
Approximately 1–2 years	Hypogammaglobulinemia was diagnosed at 20 months, and IVIG therapy was initiated.	Speech and language therapy began at approximately 1 year. Rolling re-emerged, and tablet-based AAC and purposeful communication were introduced at approximately 2 years.	Medical records and parental developmental and rehabilitation history
May 2022	Initial trio WES identified SHMT2 variants of uncertain significance and a de novo 16p11.2 microdeletion involving KAT8, without a definitive molecular diagnosis.	Ongoing multidisciplinary rehabilitation.	Genetic reports and medical records
16 February–26 March 2024	Cannabidiol was initiated at 0.3 mL once daily and titrated to 0.6 mL twice daily (10.16 mg/kg/day). Clonazepam was replaced with clobazam 2.5 mg twice daily.	Ongoing routine rehabilitation.	Medical records and parental rehabilitation history
May 2024	Trio WES reanalysis identified two heterozygous SHQ1 variants in trans, leading the clinical geneticist to consider SHQ1-related NEDDS the most likely diagnosis.	Ongoing routine rehabilitation.	Genetic reports and medical records
During 2024 follow-up	Cannabidiol reached 1.1 mL twice daily (19.4 mg/kg/day) by August and remained at this dose in November, after which an evening increase to 1.2 mL was recommended. Prolonged nocturnal tonic seizures became less frequent and subsequently ceased, while focal seizures persisted; ethosuximide was later tapered and discontinued.	Independent floor mobility through creeping was reported at approximately 4 years.	Medical records, caregiver seizure diary, and parental developmental history
13 March 2025	Clobazam and N-desmethylclobazam concentrations were within the laboratory therapeutic ranges. Follow-up liver-function tests remained within reference ranges. Increased salivation was the only early adverse event recorded; later follow-up documented no adverse events.	Ongoing routine rehabilitation.	Medical records
Approximately 5 years; November 2025	Repeat trio WES confirmed both SHQ1 variants in trans and did not identify an alternative molecular explanation.	Quadruped positioning, supported kneeling, and pulling to stand or standing with assistance were reported at approximately 5 years.	Genetic reports, medical records, and parental developmental history
22 January 2026	Neurological and physiotherapeutic functional assessment was performed.	Vojta-derived Locomotion Stages were documented at 5 years and 4 months.	Direct neurological and physiotherapeutic assessment
7 February 2026	An adapted descriptive item-level Bayley-4 assessment was performed.	Bayley-4 findings were documented at 5 years and 4 months.	Report from the certified Bayley-4 therapist
September 2025–September 2026	No prospectively standardized longitudinal outcome measurements were available.	Physical therapy was generally provided two to three times weekly for approximately 45 min. The current schedule included sessions combining NDT/Bobath with CME and one separate weekly Vojta therapy session. Speech and language therapy was provided once weekly for approximately 45–60 min. AAC was used daily; additional multidisciplinary services were regular or intermittent.	Parental rehabilitation history obtained during manuscript revision
8 September 2026	No treatment change was inferred from this single assessment.	The parents independently completed the online English-language Content-Balanced PEDI-CAT at 5 years and 11 months.	PEDI-CAT report and parental completion

Note: The neurological and physiotherapeutic assessment was performed on 22 January 2026, followed by the separate Bayley-4 assessment on 7 February 2026. The parent-reported PEDI-CAT was completed on 8 September 2026. Rehabilitation history, including the September 2025–September 2026 period shown in the table, was reconstructed retrospectively from parental reports obtained during manuscript revision and does not represent prospective follow-up. These assessments describe different aspects of functioning at different time points and do not constitute repeated measurements of treatment response. The timing of developmental milestones was also reconstructed retrospectively from parental history. The timeline describes temporal order and does not imply that medication or rehabilitation caused a subsequent clinical or functional change.

## Data Availability

The data presented in this report are contained within the article; additional patient-level data are not publicly available because of privacy restrictions.

## Supplementary Materials

The following supporting information can be downloaded at: https://www.mdpi.com/article/10.3390/jcm15197379/s1, File S1. CARE Checklist for the SHQ1 related NEDDS case report.
