# Supplementary material for: Motor, Postural, and Communicative Phenotype in SHQ1-Related Neurodevelopmental Disorder with Dystonia and Seizures: An Interdisciplinary Case Report and Hypothesis-Generating Physiotherapy Framework
Source: J Clin Med. 2026 Sep 23;15(19):7379. doi: 10.3390/jcm15197379 (PMC13665282; doi:10.3390/jcm15197379)
Supplement: Supplementary file 1 [file jcm-15-07379-s001.zip › jcm-4513525-Supplementary File S1.pdf]

CARE Checklist for the SHQ1 related NEDDS case report

Topic	Item	Checklist item description	Reported on line
**Title**	1	The diagnosis or intervention of primary focus followed by the words ‘case report’	2–5
**Key Words**	2	2 to 5 key words identifying diagnoses or interventions, including ‘case report’	56–58
**Abstract**	3a	Introduction: what is unique and what the case adds to the literature	19–22
	3b	Main symptoms and/or important clinical findings	23–48
	3c	Main diagnoses, therapeutic interventions, and outcomes	23–48
	3d	Conclusion and main take-away lesson(s)	49–55
**Introduction**	4	Why the case is unique	61–90
**Patient Information**	5a	De-identified patient-specific information	95–103; 732–734
	5b	Primary concerns and symptoms	95–175; 201–374
	5c	Medical, family, and psychosocial history, including genetic information	95–175
	5d	Relevant past interventions and outcomes	107–146; 377–447
**Clinical Findings**	6	Significant physical examination and clinical findings	201–374
**Timeline**	7	Historical and current information organized as a timeline	95–175; 448–460
**Diagnostic Assessment**	8a	Diagnostic testing: examination, laboratory testing, imaging, and assessments	95–374
	8b	Diagnostic challenges	146–175; 310–352
	8c	Diagnosis, including alternatives considered	151–175
	8d	Prognosis, where applicable	N/A
**Therapeutic Intervention**	9a	Types of therapeutic intervention	107–146; 377–447
	9b	Administration: dosage, frequency, intensity, and duration	107–146; 383–437
	9c	Changes in therapeutic intervention and rationale	109–132; 383–447
**Follow-up and Outcomes**	10a	Clinician- and caregiver-assessed outcomes	121–139; 201–374
	10b	Important follow-up diagnostic and other test results	133–198; 310–374
	10c	Intervention adherence and tolerability and how assessed	133–137; 383–437
	10d	Adverse and unanticipated events	134–137
**Discussion**	11a	Scientific discussion of strengths and limitations	462–699
	11b	Discussion of relevant medical literature with references	462–699
	11c	Scientific rationale for conclusions, including possible causes	462–699
	11d	Primary take-away lessons in a one-paragraph conclusion	701–709
**Patient Perspective**	12	Patient or family perspective on the treatment received	Not reported
**Informed Consent**	13	Consent for publication, including anonymized photographic material	732–734 — Yes

CARE adherence statement: lines 92–93. The completed checklist is identified in the manuscript as Supplementary File S1. Line references correspond to the clean manuscript “jcm-4513525_minor_changes_clear.docx” and its accompanying 20-page PDF.
